# The Expanded Bead Size of Corneal C-Nerve Fibers Visualized by Corneal Confocal Microscopy Is Associated with Slow Conduction Velocity of the Peripheral Nerves in Patients with Type 2 Diabetes Mellitus

**DOI:** 10.1155/2016/3653459

**Published:** 2016-08-03

**Authors:** Fukashi Ishibashi, Rie Kojima, Miki Taniguchi, Aiko Kosaka, Harumi Uetake, Mitra Tavakoli

**Affiliations:** ^1^Ishibashi Clinic, 1-9-41-2 Kushido, Hatsukaichi, Hiroshima 738-0033, Japan; ^2^Centre for Endocrinology and Diabetes, Institute of Human Development, University of Manchester, 46 Grafton Street, Manchester M13 9NT, UK; ^3^University of Exeter Medical School, St Luke's Campus, South Cloisters, Exeter EX1 2LU, UK

## Abstract

This study aims to establish the corneal nerve fiber (CNF) morphological alterations in a large cohort of type 2 diabetic patients and to investigate the association between the bead size, a novel parameter representing composite of accumulated mitochondria, glycogen particles, and vesicles in CNF, and the neurophysiological dysfunctions of the peripheral nerves. 162 type 2 diabetic patients and 45 healthy control subjects were studied in detail with a battery of clinical and neurological examinations and corneal confocal microscopy. Compared with controls, patients had abnormal CNF parameters. In particular the patients had reduced density and length of CNF and beading frequency and increased bead size. Alterations in CNF parameters were significant even in patients without neuropathy. The HbA1c levels were tightly associated with the bead size, which was inversely related to the motor and sensory nerve conduction velocity (NCV) and to the distal latency period of the median nerve positively. The CNF density and length positively correlated with the NCV and amplitude. The hyperglycemia-induced expansion of beads in CNF might be a predictor of slow NCV in peripheral nerves in type 2 diabetic patients.

## 1. Introduction

Diabetic neuropathy is a major complication of diabetes that may affect the sensory, autonomic, and motor nerves [1]. Mitochondria play a pivotal role in controlling nerve function by their fusion, fission, and trafficking [2, 3] and their morphological and functional anomalies play a pivotal role in developing diabetic neuropathy [4, 5]. Corneal confocal microscopy (CCM) has been used to diagnose small C-fiber neuropathy [6, 7] and visualizes composites of the accumulated mitochondria, glycogen particles, and vesicles [8] as a bead [9]. In diabetic mice the presence of collections of small mitochondria and the formation of markedly enlarged mitochondria has been reported by Schmidt et al. [10]. Using IENFs of patients with diabetic neuropathy, Hamid et al. [4] observed larger mitochondrial signals composed of clustered smaller, potentially dysfunctional mitochondria or of larger predegenerative mitochondria due to a disruption in axonal transport. In small fiber neuropathy patients with relatively preserved IENFs, mitochondrial loss as judged by the significant reduction of mitochondrial oxidative phosphorylation was found [11]. However, there has been no report to associate the bead size (BS) of corneal nerve fibers (CNFs) and dysfunctions of the peripheral nerves in diabetic patients, because the size of an individual bead is too small to be assessed. We already reported that enlarging and smoothing of beads by S-Spline Max algorithm make it feasible to determine the size of an individual bead [12].

The aim of the present study is to associate the BS in CNFs determined by subsequent image processing with the neurophysiological dysfunctions of the peripheral nerves in patients with type 2 diabetes, elucidating whether the BS of CNFs might predict dysfunctions in the peripheral nerves.

## 2. Research Design and Methods

### 2.1. Subjects

162 Japanese patients with type 2 diabetes and 45 healthy control subjects (HbA1c < 5.7% (38.8 mmol/mol), fasting plasma glucose < 5.5 mmol/L or casual postprandial plasma glucose < 7.7 mmol/L) were enrolled in this study at the period between May 2013 and January 2015 at the Ishibashi Clinic, Hiroshima, Japan. Subjects were excluded from the study if they consumed alcohol > one unit/day, had neurological symptoms due to neurological disorders other than diabetic neuropathy or had been diagnosed with the neuropathy due to another cause, or wore contact lenses or had history of refractive surgery or anterior segment trauma. The healthy volunteers were recruited from the general population. Written informed consent was obtained according to the Declaration of Helsinki. The Ethics Committee of the Ishibashi Clinic approved the protocol of the present research.

### 2.2. Laboratory Data

The HbA1c levels were converted to National Glycohemoglobin Standardization Program (NGSP) units by adding 0.4% to the measured values [13] and subsequently converted to the International Federation of Clinical Chemistry values by using the equation [(10.93NGSP) − 23.50]. The serum creatinine, lipid levels, the urinary albumin/creatinine ratio (ACR), and the estimated glomerular filtration rate (eGFR) were also determined.

### 2.3. Neurophysiological Examinations

Electrophysiology and nerve conduction velocity (NCV) studies were performed using an electromyography instrument (NeuroPak S1, NIHON KOHDEN, Tokyo, Japan). The motor conduction velocity (MCV, median nerve) and sensory conduction velocity (SCV, ulnar and sural nerve), action potential amplitudes of these three nerves, and the distal latency period of the median nerve in the wrist segment were determined.

The vibration perception threshold (VPT) was measured at the left medial malleolus using a biothesiometer (Biomedical Instruments, Newbury, OH, USA). The average of eight readings has been included. The warm and cold perception thresholds (PTs) at the dorsum of the foot were determined using a thermal stimulator that was controlled by a Peltier element (Intercross-200, Intercross Co., Tokyo, Japan). To assess the cardiovagal function of the autonomic nervous system, the coefficient of variation of R-R intervals (CV_R-R_) was calculated from the R-R intervals of 200 samples on an electrocardiogram.

### 2.4. Assessment of Neuropathy

Severity of neuropathy and neurological deficits were assessed using the Neuropathy Disability Score (NDS) [14] which includes evaluation of vibration, pin prick, and temperature perception as well as the presence or absence of ankle reflexes to establish the severity of neuropathy: NDS 0–2, no neuropathy; NDS 3–5, mild neuropathy; NDS 6–8, moderate neuropathy; and NDS 9-10, severe neuropathy.

### 2.5. CCM

All study subjects were examined using Heidelberg Retina Tomograph (HRT III, Heidelberg Engineering, Heidelberg, Germany) [9]. The technicians who were performing CCM examination were blinded to patient groups and the signs and symptoms of patients. The examined eye was anesthetized by instilling one drop of 0.4% benoxinate hydrochloride (Santen Pharmaceutical Co., Osaka, Japan). Comfort Gel (Dr. Mann Pharma, Berlin, Germany) was applied to the lens, and a disposable sterilized TomoCap (Heidelberg Engineering GmbH, Dossenheim, Germany) was mounted on the holder to cover the objective lens. After applying Comfort Gel to the TomoCap, the lens was slowly advanced until the gel touched the cornea. More than 50 images of the subbasal nerve plexus were captured using a section mode, and we analyzed at least 6 high-clarity images per subject for the quantification of the following parameters to define changes in the CNFs: (1) CNF density (CNFD): the total number of major nerve fibers/mm^2^ of corneal tissue; (2) CNF length (CNFL): the total length of all nerve fibers (mm/mm^2^); (3) corneal nerve branch density (CNBD): the number of branches emanating from all major nerve trunks/mm^2^ of corneal tissue; (4) tortuosity grade (TG); (5) frequency/0.1 mm of beading (BF); and (6) BS determined after enlarging 5 times and smoothing the original image of CCM using S-Spline Max algorithm (PhotoZoom Pro 4, Gungle Inc., Tokyo, Japan). The pixel numbers of 120 beads were counted using Photoshop Elements 8.0 (Adobe Systems Inc., San Jose, CA, USA) and averaged.

Except for the TG and BS, all measurements were performed using ImageJ (Texelcraft, Tokyo, Japan); the TG was measured by the grading system of Oliveira-Soto and Efron [15].

### 2.6. Statistical Analyses

The post hoc analysis of sample power revealed that, with the use of a one-sided ANOVA (significance of 0.05) and Kruskal-Wallis test, the present study population provided statistical power ranged from 0.82 to 0.99. All statistical analyses were performed using the SPSS medical package (SPSS version 19, Chicago, IL, USA). All values are presented as the mean ± SEM. All data sets were tested for the normality using the Shapiro-Wilk test. For normally distributed variables, the comparisons between controls and total diabetic patients or subgroups stratified by NDS were made using one-way ANOVA (for continuous variables) and the *χ* ^2^-test (for categorical variables) followed by Bonferroni corrections. For nonnormally distributed variables, the Kruskal-Wallis test was applied with subsequent Mann-Whitney *U* test and Bonferroni corrections. The correlations between the CNF measures and the clinical factors or the results of neurophysiological tests were assessed by the Spearman rank correlation test. The sensitivity and specificity of CCM measures in differentiating between control subjects and patients without neuropathy or between patients without neuropathy and with neuropathy were assessed using receiver operating characteristic (ROC) analysis. *p* < 0.05 was considered significant.

## 3. Results

### 3.1. Characteristics of Healthy Controls and Patients with Type 2 Diabetes

The clinical characteristics and detailed assessment of diabetic neuropathy in diabetic patients and controls are summarized in Table 1. The gender ratio and age were similar between controls, total diabetic patients, and their subgroups. The BMI of all patients and the mild neuropathy subgroup was higher than that in controls. The blood pressure in all patients was higher than that in control subjects. The angiotensin receptor blocker or angiotensin-converting enzyme inhibitor was prescribed more frequently for the total patient group and the moderate neuropathy subgroup than for controls. The HbA1c levels in the total patient group and all diabetic subgroups were higher than those in controls, while no difference was found between diabetic subgroups. The LDL-cholesterol level in the total patient group and a mild neuropathy subgroup was elevated compared with that in controls. Statins were prescribed more frequently for the total diabetic patients than for the controls. The HDL-cholesterol level in the total patient group and patients without neuropathy and with moderate neuropathy was lower than that in controls. The triglycerides level in the total patient group and subgroups with mild or moderate neuropathy was increased compared with that in controls. The ACR in the whole patient group and all diabetic subgroups was significantly higher than controls. There were significant differences in the mean NDS between all subgroups of diabetic patients stratified by NDS.

### 3.2. Neurological Examinations

Except for the MCV of the median nerve, there existed no difference in the neurophysiological tests between controls and the subgroup without neuropathy. The MCV, amplitude, and distal latency period of the median nerve and the SCV and amplitude of ulnar and sural nerve deteriorated relative to the severity of neuropathy (Table 1). VPT increased relative to the severity of neuropathy. CV_R-R_ and temperature PTs in the moderate neuropathy subgroup were impaired compared with those in controls or patients without neuropathy (Table 1).

### 3.3. Corneal Nerves Morphological Parameters in Control Subjects and Type 2 Diabetic Patients

**Figure 1:**
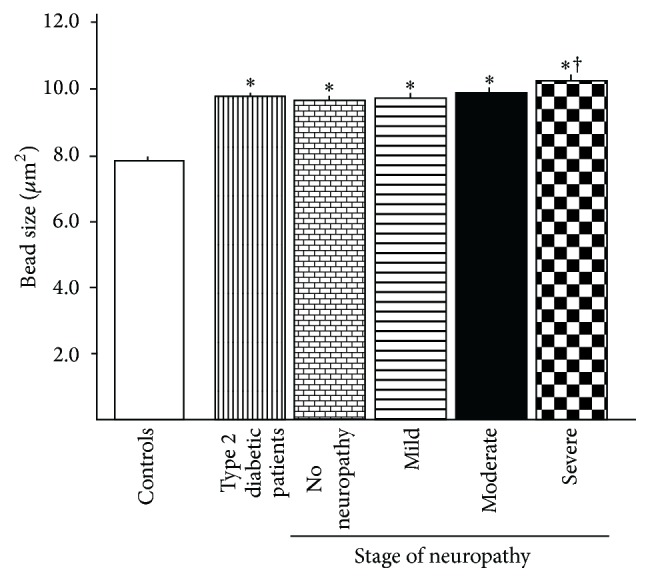
Comparison of the bead sizes of the corneal nerve fibers between control subjects, total type 2 diabetic patients, and diabetic subgroups stratified by the severity of neuropathy. Data are the mean ± SEM. ^*∗*^ *p* < 0.001 compared with control subjects and ^†^ *p* < 0.05 compared with patients without neuropathy.

In the cohort of patients without neuropathy, the CNFD, CNFL, CNBD, and BF were markedly reduced, and TG and BS were increased compared with controls. The further mild deterioration of CNFD and CNFL was found in the moderate neuropathy subgroup and BS in patients with severe neuropathy (Table 2). BS in patients without neuropathy expanded compared with that of controls and further expansion was found only in patients with severe neuropathy (Figure 1).

**Figure 2:**
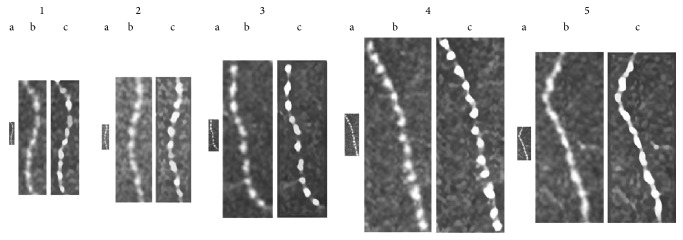
The representative beads in the original CCM images (a), images simply magnified 5 times by Photoshop (b), and images enlarged 5 times with smoothing by the S-Spline Max algorithm (c) in a control subject (1, male, 56 years, NDS: 0), patient without neuropathy (2, male, 52 years, NDS: 1), patient with mild neuropathy (3, male, 54 years, NDS: 5), patient with moderate neuropathy (4, male, 56 years, NDS: 7), and patient with severe neuropathy (5, male, 57 years, NDS: 9).

Figure 2 illustrated the representative beads in original CCM images (a), when the images were simply magnified 5 times by Photoshop (b), and when the images were enlarged 5 times with smoothing by the S-Spline Max algorithm (c) in a control subject (1), a patient without diabetic neuropathy (2), a patient with mild diabetic neuropathy (3), a patient with moderate diabetic neuropathy (4), or a patient with severe diabetic neuropathy (5). The images that were simply magnified by Photoshop were not suitable to determine the pixel numbers accurately. In comparison, the images processed by the S-Spline Max algorithm were clearly demarcated, and the pixel numbers were easily determined. Compared with the BS of controls, those in diabetic patients appeared to expand. The intrarater variability (averaged CV) in the measurement of the pixel numbers of bead in 10 control subjects was 12.7 ± 2.6%.

**Figure 3:**
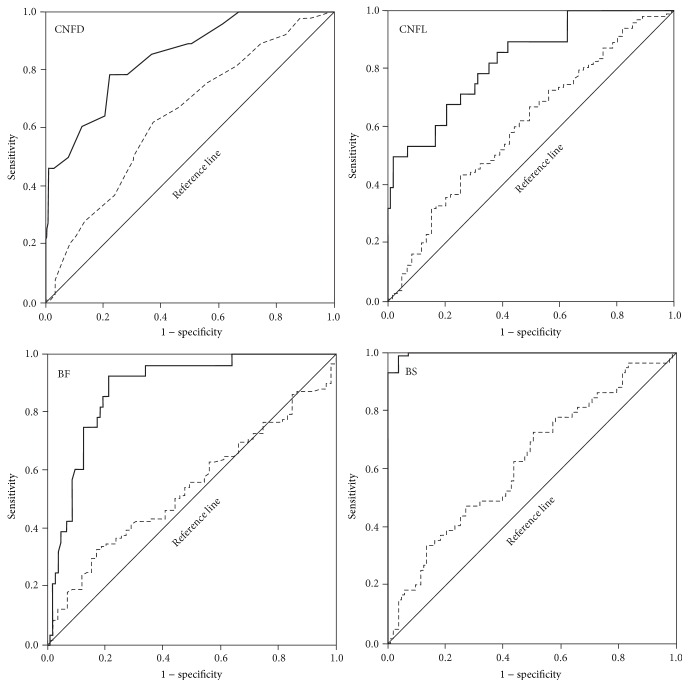
Receiver operating characteristic (ROC) curve analyses for CNFD, CNFL, BF, and BS between control subjects and patients without neuropathy (NDS < 3) (—) and between patients without neuropathy (NDS < 3) and with neuropathy (NDS > 3) (- - -).

According to the ROC curves for the four CCM parameters between controls and patients without neuropathy (NDS < 3) (Figure 3 and Table 3), AUC was 0.847 for CNFD, 0.830 for CNFL, 0.883 for BF, and 0.997 for BS. Because the AUC, sensitivity, and specificity of BS are the best among parameters of CCM, it is considered that BS is the most reliable marker of CNF alteration in type 2 diabetes before developing neuropathy (Table 3). On the other hand, AUC between patients without neuropathy (NDS < 3) and with neuropathy (NDS > 3) did not seem to be useful in detecting the presence of neuropathy: AUC was 0.610 for CNFD, 0.585 for CNFL, 0.538 for BF, and 0.602 for BS (Table 3).

### 3.4. Correlations between CCM Parameters and Clinical Factors or Neurophysiological Tests

There was significant correlation between HbA1c and all CNF parameters except TG and BF. NDS was associated with CNFD and CNFL inversely and with BS positively (Table 4). The BS had the robust inverse association with MCV and the positive association with the distal latency period of the median nerve and inversely related with the SCV of the ulnar and sural nerve. On the other hand, CNFD and CNFL had the modest association with the NCV as well as the amplitudes of the peripheral nerves. The weak but significant relationships between the CNFD, CNFL, and CNBD and CV_R-R_ or cold PT were found, while BS had the positive relationship with a VPT (Table 4).

## 4. Discussion

Diabetic neuropathy is a major complication of diabetes and leads to significant morbidity and mortality [16]. The chronic hyperglycemia-induced changes in mitochondrial structure, function, and dynamics may lead to the development of diabetic neuropathy [2, 3, 17]. The increase in the number of small mitochondria due to excessive fission in the dorsal root fibers of db/db mouse with diabetic neuropathy has been reported [18]. In a study with Akita diabetic mouse with diabetic neuropathy, sympathetic ganglia show the accumulation of minute mitochondria [19]. In patients with diabetic neuropathy the larger mitochondrial signals composed of clustered smaller, potentially dysfunctional mitochondria or of larger predegenerative mitochondria in IENFs due to a disruption in axonal transport were observed [4]. In the small fiber neuropathy patients in early stage, mitochondrial loss was reported [11]. Since an invasive skin or nerve biopsy is mandatory for the morphological assessment of mitochondrial number and size, it could not be performed as a clinical routine procedure.

To our knowledge, this is the largest cohort of type 2 diabetic patients that have been studied in detail for standard clinical examinations and CCM, and the new parameters including BS have been studied for the first time.

The CNF pathology assessed by CCM has been proposed as a surrogate marker for a small fiber neuropathy in patients with diabetes [6, 7], because the ROC curves for the CCM parameters (CNFD, CNFL, and CNBD) for NDS > 3 revealed good AUC, sensitivity, and specificity [6] and because the CCM analysis using a conventional and novel algorithm to reconstruct CNF images had revealed the significant correlations between CNFD, CNFL, or CNBD and the NCV, VPT, temperature PT, heart rate variability, or the clinical severity of diabetic neuropathy [20]. The ROC analysis of CCM parameters in the present study revealed that the AUC, sensitivity, and specificity of CCM measures in differentiating between control subjects and patients without neuropathy (NDS < 3) were excellent and among the parameters of CNF morphology, BS had the best AUC, sensitivity, and specificity.

Using the thin section of a freshly isolated human cornea the electron microscopy can identify beads in CNFs as a composite of the accumulated mitochondria, glycogen particles, and vesicles [8, 21] playing some role in controlling CNF functions [21] with similar ultrastructure to nociceptor terminals. The present study revealed that BF decreased and BS expanded in patients with diabetes compared with control subjects. In the present study BS inversely associated with CNFD (*r* = −0.269, *p* < 0.001), CNFL (*r* = −0.212, *p* = 0.003), and CNBD (*r* = −0.229, *p* = 0.001), and BF had positive relationship with CNFD (*r* = 0.252, *p* < 0.001), CNFL (*r* = 0.205, *p* = 0.005), and CNBD (*r* = 0.184, *p* = 0.011). Therefore, altered beading structures may have relationship with the loss of main nerve fibers as well as branches. Although the increased irregularity in the periodicity of CNF beading has been reported in streptozotocin- (STZ-) diabetic rat [22], alteration in the BS and in composition of bead remained to be clarified. The axonal trafficking of mitochondria is controlled by cytoskeletons, motor proteins, and ATP fuel supply [23]. In STZ-diabetic rats, axonal neurofilaments in peripheral nerve were deleted [24], and sciatic level of kinesin 5B motor protein which is involved in the axonal transport of mitochondria is changed [25]. These results suggested that changes in the distribution of mitochondria in CNFs might occur resulting in altered number and size of beads. However, we could not determine the exact mitochondrial area in bead using mitochondria marker, because the present study was an in vivo human study using CCM. Therefore, the contribution of mitochondrial number and size to the BS remained to be determined. In patients with diabetic neuropathy [26] and the spontaneously diabetic BB-Wistar-rat [27], mitochondrial accumulation of glycogen particles was observed. In experimental diabetic animals, the loss of vesicles in presynapse [28] and autonomic nerve endings [29] was reported. Therefore, the alterations in components of bead other than mitochondria might influence the density and size of bead.

The present research found the significant relationships between parameters of main CNF and branch and the NCV and amplitudes of the peripheral nerves, heart rate variability, and a cold PT. Although we and others previously reported the preliminary findings of lower BF of CNFs in diabetic patients [30–32], the relationship between the BS and neurophysiological tests had never been investigated. The beads can be clearly demonstrated along CNFs by CCM. However, the individual bead in the original CCM image is too small for assessing its area. The enlarging and smoothing of the original CCM image by S-Spline Max algorithm enable us to determine the size of an individual bead in CNFs in spite of the uncertain accuracy and exactness of this method.

Based on the current results, CCM can detect CNF alteration in patients without clinical evidence of neuropathy compared with those of the controls. According to ROC analysis BS had the largest AUC with the best sensitivity and specificity compared with CNFD, CNFL, and BF. These results indicated that the diabetes-induced expansion of BS and changes in other morphological parameters of CCM might have predictive value for the dysfunctions of the peripheral nerves. The expansion of beads occurred in patients without the neuropathy, getting larger in patients with severe neuropathy. The BS had good negative relationship with MCV and SCV of the peripheral nerves and directly associated with a distal latency period of the median nerve. In the present study the BS correlated strongly to HbA1c levels. In myelinated murine axon, the oxidative stress alters the external morphology and reduces the transport of mitochondria at the nodes of Ranvier. These mitochondrial changes expand from the node of Ranvier bidirectionally [33]. In STZ-diabetic mice, there is a significant increase in synaptic delay compared with control mice, and the accumulation of degenerated mitochondria of presynaptic axon of the neuromuscular junction was observed [34]. However, because there has been no report investigating the morphological changes in mitochondria of CNFs in patients with diabetes, we could not assume that the bead in the CNFs is a surrogate marker of the size of mitochondrial area in the peripheral nerves in diabetic patients.

We acknowledge limitations to the present study, which may affect the interpretation of the results. First, although we measured the BS of CNF in patients with type 2 diabetes, we did not determine whether the number of mitochondria in an expanded bead in CNFs increased or not. Furthermore, alteration in other components of bead could influence BS. Second, we assessed BS after enlarging and smoothing of original bead. However, the accuracy and preciseness of this method were not established. The future improvement in a resolution of CCM apparatus will make it possible to assess the BS directly using an original CCM image. Lastly, the potential bias determining BF and BS by human errors could not be ruled out. The automatic analyzing system for beading as already developed for other corneal morphological parameters of CNFs [35] would eliminate this type of errors.

## 5. Conclusions

In conclusion, the expansion of beads of the CNFs in patients with type 2 diabetes occurred before the development of neuropathy and was related to the slow NCV and the prolonged distal latency period of the peripheral nerves. The BS in the CNFs has a predictive value for developing the slow NCV of the peripheral nerves in patients with type 2 diabetes. However, elucidating the mechanisms of the expansion of bead in CNFs was beyond the scope of the present study.

## Figures and Tables

**Table 1:** Clinical characteristics and neurophysiological test of the control subjects, type 2 diabetic patients, and their subgroups stratified by the stages of neuropathy.

	Control subjects	Type 2 diabetic patients				
		All type 2 diabetic patients	Stages of neuropathy			
			No neuropathy NDS (0–2)	Mild NDS (3–5)	Moderate NDS (6–8)	Severe NDS (9-10)
Number (M/F) (%)	45 (27/18) (60/40)	162 (106/56) (65.4/34.6)	47 (29/18) (61.7/38.3)	56 (37/19) (66.1/33.9)	46 (31/15) (67.4/32.6)	13 (9/4) (69.2/30.8)
Age (years)	52.8 ± 0.7	53.1 ± 0.8	52.4 ± 1.4	52.0 ± 1.4	54.2 ± 1.4	56.9 ± 2.5
Duration of diabetes (years)		6.7 ± 0.5	5.0 ± 0.8	6.4 ± 0.7	8.1 ± 1.1	9.2 ± 1.4
BMI (kg/m^2^)	23.2 ± 0.6	25.2 ± 0.3^*∗*^	25.2 ± 0.6	25.2 ± 0.5^†^	25.1 ± 0.7	25.8 ± 1.2
SBP (mmHg)	134.4 ± 2.2	153.0 ± 1.8^*∗*^	149.9 ± 3.3^*∗*^	152.3 ± 2.8^*∗*^	155.7 ± 3.9^*∗*^	158.5 ± 7.8^‡^
DBP (mmHg)	79.8 ± 0.9	90.4 ± 0.9^*∗*^	90.6 ± 1.7^*∗*^	90.3 ± 1.4^*∗*^	90.5 ± 1.6^*∗*^	90.1 ± 3.0^†^
Number treated with ARB/ACEI (%)	2 (4.4)	26 (16.0)^†^	8 (17.0)	7 (12.5)	10 (21.7)^†^	1 (7.7)
HbA1c (NGSP, %)	5.5 ± 0.04	8.7 ± 0.20^*∗*^	8.5 ± 0.34^*∗*^	9.0 ± 0.32^*∗*^	8.4 ± 0.27^*∗*^	9.8 ± 0.64^*∗*^
HbA1c (mmol/mol)	36.5 ± 0.41	69.4 ± 1.80	67.1 ± 3.48	72.0 ± 3.29	65.8 ± 2.81	79.6 ± 6.51
LDL-C (mmol/L)	3.16 ± 0.11	3.58 ± 0.08^‡^	3.54 ± 0.16	3.79 ± 0.13^†^	3.34 ± 0.14	3.69 ± 0.31
Number treated with statins (%)	2 (4.4)	31 (19.1)^†^	10 (21.3)	11 (19.6)	8 (17.4)	2 (15.4)
HDL-C (mmol/L)	1.77 ± 0.072	1.47 ± 0.059^*∗*^	1.38 ± 0.056^*∗*^	1.59 ± 0.156	1.43 ± 0.064^‡^	1.45 ± 0.096
Triglycerides (mmol/L)	1.45 ± 0.15	2.26 ± 0.12^*∗*^	2.19 ± 0.24	2.34 ± 0.19^‡^	2.27 ± 0.23^†^	2.20 ± 0.36
ACR (mg/gCr)	8.0 ± 1.6	99.8 ± 35.2^*∗*^	58.9 ± 23.1^‡^	49.6 ± 15.2^*∗*^	176.5 ± 118.4^*∗*^	192.5 ± 78.9^*∗*^
eGFR (mL/min)	80.8 ± 2.3	83.5 ± 1.4	84.3 ± 2.7	85.4 ± 2.2	82.3 ± 2.7	77.3 ± 4.6
Mean NDS value	0	4.36 ± 0.22^*∗*^	0.87 ± 0.12^*∗*^	4.16 ± 0.10^*∗*,§^	6.76 ± 0.12^*∗*,װ^	9.38 ± 0.14^*∗*,¶^
Neurophysiological test						
MCV of MN (m/sec)	57.9 ± 0.44	53.7 ± 0.36^*∗*^	54.8 ± 0.70^‡^	53.6 ± 0.62^*∗*^	52.4 ± 0.62^*∗*^	48.2 ± 1.10^*∗*,§,װ,#^
Amplitude of MN (mV)	7.77 ± 0.37	6.00 ± 0.16^*∗*^	6.90 ± 0.26	6.16 ± 0.28^†^	5.31 ± 0.28^*∗*,*∗∗*^	4.92 ± 0.81^*∗*,*∗∗*^
Distal latency of MN (msec)	3.16 ± 0.06	3.69 ± 0.053^*∗*^	3.54 ± 0.11	3.71 ± 0.09^*∗*^	3.79 ± 0.095^*∗*^	3.79 ± 0.14^‡^
SCV of ulnar nerve (m/sec)	64.0 ± 0.62	59.5 ± 0.37^*∗*^	61.2 ± 0.57	60.0 ± 0.58^*∗*^	58.4 ± 0.73^*∗*,††^	55.8 ± 1.40^*∗*,*∗∗*,‡‡^
Amplitude of ulnar nerve (*μ*V)	29.5 ± 2.3	19.6 ± 0.92^*∗*^	22.7 ± 2.0	21.7 ± 1.5	16.0 ± 1.3^*∗*^	12.2 ± 1.8^*∗*,††^
SCV of sural nerve (m/sec)	48.2 ± 0.39	45.7 ± 0.21^*∗*^	47.1 ± 0.33	45.7 ± 0.34^*∗*^	45.0 ± 0.42^*∗*,*∗∗*^	43.2 ± 0.55^*∗*,§,‡‡^
Amplitude of sural nerve (*μ*V)	12.5 ± 0.76	10.8 ± 0.58^*∗*^	10.6 ± 0.42	10.3 ± 0.29	9.2 ± 0.34^*∗*^	8.8 ± 0.70^‡^
Vibration PT (*μ*/120 c/s)	2.00 ± 0.29	3.61 ± 0.25^*∗*^	2.48 ± 0.26	3.21 ± 0.32	3.88 ± 0.55^‡^	8.48 ± 0.99^*∗*,§,װ,¶^
CV_R-R_ (%)	3.71 ± 0.15	3.38 ± 0.12^*∗*^	3.69 ± 0.24	3.54 ± 0.22	2.99 ± 0.18^†^	2.91 ± 0.40
Warm PT (W/m^2^)	−554 ± 16.4	−628 ± 20.5^‡^	−545 ± 22.0	−612 ± 31.0	−652 ± 27.2^†^	−915 ± 160^*∗*,§^
Cold PT (W/m^2^)	487 ± 16.5	573 ± 14.3^*∗*^	520 ± 19.2	565 ± 17.0	585 ± 19.2^†,*∗∗*^	769 ± 119^*∗*,§^

Data are the mean ± standard error of the mean in control subjects, type 2 diabetic patients, and their subgroups stratified by the stages of the neuropathy according to the Neuropathy Disability Score (NDS) [14]. ^*∗*^ *p* < 0.001 compared with control subjects, ^†^ *p* < 0.05 compared with control subjects, ^‡^ *p* < 0.01 compared with control subjects, ^§^ *p* < 0.001 compared with patients without neuropathy, ^װ^ *p* < 0.001 compared with patients with mild neuropathy, ^¶^ *p* < 0.001 compared with patients with moderate neuropathy, ^#^ *p* < 0.05 compared with patients with moderate neuropathy, ^*∗∗*^ *p* < 0.01 compared with patients without neuropathy, ^††^ *p* < 0.05 compared with patients without neuropathy, and ^‡‡^ *p* < 0.05 compared with patients with mild neuropathy.

ACEI, angiotensin-converting enzyme inhibitor; ACR, albumin/creatinine ratio; ARB, angiotensin receptor blocker; BMI, body mass index; DBP, diastolic blood pressure; eGFR, estimated glomerular filtration rate; HDL-C, high density lipoprotein-cholesterol; LDL-C, low density lipoprotein-cholesterol; MN, median nerve; PT, perception threshold; SBP, systolic blood pressure.

**Table 2:** Summary of corneal nerves morphological parameters in control subjects, type 2 diabetic patients, and their subgroups stratified by the stages of neuropathy.

Corneal nerves morphological parameters	Control subjects	Patients with type 2 diabetes				
		All type 2 diabetic patients	Stratified by stages of neuropathy			
			No neuropathy NDS (0–2)	Mild NDS (3–5)	Moderate NDS (6–8)	Severe NDS (9-10)
Corneal nerve fiber density (CNFD) (no/mm^2^)	33.0 ± 1.2	23.3 ± 0.41^*∗*^	25.3 ± 0.72^*∗*^	23.5 ± 0.66^*∗*^	21.7 ± 0.83^*∗*,†^	21.6 ± 1.13^*∗*^
Corneal nerve fiber length (CNFL) (mm/mm^2^)	15.5 ± 0.53	9.5 ± 0.16^*∗*^	12.3 ± 0.31^*∗*^	11.7 ± 0.30^*∗*^	11.0 ± 0.38^*∗*,†^	11.3 ± 0.49^*∗*^
Corneal nerve branch density (CNBD) (no/mm^2^)	14.5 ± 1.2	9.6 ± 0.26^*∗*^	9.5 ± 0.39^‡^	10.0 ± 0.51^‡^	9.3 ± 0.54^‡^	9.1 ± 0.67^§^
Tortuosity grade (TG)	1.98 ± 0.06	2.49 ± 0.03^*∗*^	2.47 ± 0.05^*∗*^	2.51 ± 0.05^*∗*^	2.47 ± 0.05^*∗*^	2.54 ± 0.13^*∗*^
Beading frequency (BF) (number/0.1 mm)	23.7 ± 0.29	20.4 ± 0.16^*∗*^	20.1 ± 0.27^*∗*^	20.9 ± 0.32^*∗*^	20.3 ± 0.27^*∗*^	19.6 ± 0.36^*∗*^
Bead size (BS) (*μ*m^2^)	7.94 ± 0.07	9.84 ± 0.05^*∗*^	9.65 ± 0.09^*∗*^	9.82 ± 0.08^*∗*^	9.95 ± 0.09^*∗*^	10.3 ± 0.17^*∗*,†^

Data are expressed as mean ± standard error of the mean (SEM) in control subjects, type 2 diabetic patients, and their subgroups stratified by the Neuropathy Disability Score (NDS). ^*∗*^ *p* < 0.001 compared with control subjects, ^†^ *p* < 0.05 compared with patients without neuropathy, ^‡^ *p* < 0.01 compared with control subjects, and ^§^ *p* < 0.05 compared with control subjects.

**Table 3:** Differentiating efficacy of corneal nerve fiber parameters as AUC and *p* values with CCM cut-off with sensitivity and specificity between control subjects and type 2 diabetic patients without neuropathy or between type 2 diabetic patients with or without neuropathy.

Variable	Between control subjects and type 2 diabetic patients without neuropathy					Between type 2 diabetic patients without or with neuropathy				
	AUC	*p* value	Cut-off	Sensitivity	Specificity	AUC	*p* value	Cut-off	Sensitivity	Specificity
CNFD	0.847	<0.0001	28.1/mm^2^	0.79	0.78	0.610	0.020	23.1/mm^2^	0.66	0.54
CNFL	0.830	<0.0001	13.4 mm/mm^2^	0.79	0.68	0.585	0.071	11.6 mm/mm^2^	0.63	0.55
BF	0.883	<0.0001	22.1/0.1 mm	0.92	0.83	0.538	0.427	20.5/0.1 mm	0.48	0.66
BS	0.997	<0.0001	8.47 *μ*m^2^	0.99	0.96	0.602	0.031	9.76 *μ*m^2^	0.65	0.53

BF, beading frequency; BS, bead size; CNFD, corneal nerve fiber density; CNFL, corneal nerve fiber length.

**Table 4:** Relationship between the various measures of corneal nerve fibers and clinical and neurophysiological parameters in patients with type 2 diabetes.

	CNFD		CNFL		CNBD		TG		BF		Bead size	
	*β*	*p*	*β*	*p*	*β*	*p*	*β*	*p*	*β*	*p*	*β*	*p*
Gender	−0.056	0.482	−0.054	0.498	0.093	0.240	−0.040	0.617	−0.083	0.295	0.034	0.669
Age	−0.012	0.882	−0.060	0.450	−0.066	0.405	0.004	0.959	−0.026	0.744	0.057	0.475
Duration of DM	−0.094	0.236	−0.083	0.291	−0.071	0.372	0.036	0.645	−0.025	0.750	−0.040	0.617
SBP	−0.107	0.176	−0.105	0.183	0.011	0.886	−0.107	0.174	−0.010	0.902	−0.032	0.685
DBP	−0.132	0.093	−0.113	0.150	0.015	0.848	−0.139	0.079	−0.004	0.961	0.017	0.830
HbA1c	−0.200	0.011	−0.213	0.007	−0.168	0.032	0.003	0.973	−0.080	0.313	0.352	<0.0001
LDL-C	0.028	0.724	0.011	0.891	−0.007	0.925	0.075	0.343	−0.116	0.141	−0.006	0.937
HDL-C	−0.052	0.509	−0.105	0.182	−0.066	0.403	0.032	0.683	0.027	0.731	−0.043	0.586
Triglycerides	−0.038	0.633	−0.060	0.451	−0.077	0.330	0.034	0.670	−0.015	0.848	0.002	0.984
NDS	−0.255	0.001	−0.208	0.008	−0.019	0.815	0.034	0.665	−0.012	0.883	0.203	0.010
MCV of MN	0.199	0.011	0.193	0.014	0.054	0.494	0.063	0.426	0.050	0.526	−0.358	<0.0001
Amplitude of MN	0.208	0.008	0.172	0.028	−0.009	0.914	−0.083	0.292	−0.028	0.722	0.091	0.248
Distal latency of MN	−0.090	0.256	−0.090	0.256	−0.018	0.819	−0.083	0.295	0.065	0.413	0.294	<0.0001
SCV of UN	0.200	0.011	0.225	0.004	0.138	0.079	0.070	0.379	0.037	0.644	−0.273	<0.0001
Amplitude of UN	0.195	0.013	0.215	0.006	0.040	0.616	−0.031	0.699	0.053	0.504	0.083	0.294
SCV of SN	0.201	0.010	0.223	0.004	0.107	0.175	0.105	0.183	0.031	0.698	−0.237	0.002
Amplitude of SN	0.224	0.004	0.232	0.003	0.076	0.339	−0.064	0.417	0.098	0.215	0.004	0.963
Vibration PT	−0.019	0.812	0.029	0.803	0.016	0.837	0.097	0.220	−0.046	0.562	0.199	0.011
CV_R-R_	0.161	0.040	0.210	0.007	0.170	0.030	−0.026	0.744	0.009	0.909	−0.098	0.216
Warm PT	0.021	0.790	−0.002	0.985	−0.073	0.358	−0.149	0.058	−0.025	0.753	0.051	0.523
Cold PT	−0.142	0.071	−0.157	0.046	−0.015	0.853	−0.030	0.706	−0.028	0.727	0.025	0.754

BF, beading frequency; CNBD, corneal nerve branch density; CNFD, corneal nerve fiber density; CNFL, corneal nerve fiber length; CV, coefficient of variation; DBP, diastolic blood pressure; DM, diabetes mellitus; HDL-C, high density lipoprotein-cholesterol; LDL-C, low density lipoprotein-cholesterol; MCV, motor conduction velocity; MN, median nerve; NDS, Neuropathy Disability Score; PT, perception threshold; SBP, systolic blood pressure; SCV, sensory conduction velocity; SN, sural nerve; TG, tortuosity grade; UN, ulnar nerve.
